# Characterization of some *Brucella *species from Zimbabwe by biochemical profiling and AMOS-PCR

**DOI:** 10.1186/1756-0500-2-261

**Published:** 2009-12-22

**Authors:** Gift Matope, Evison Bhebhe, John Bwalya Muma, Eystein Skjerve, Berit Djønne

**Affiliations:** 1 Department of Paraclinical Veterinary Studies, University of Zimbabwe, PO Box MP 167, Mount Pleasant, Harare, Zimbabwe; 2 Department of Disease Control, University of Zambia, School of Veterinary Medicine, PO Box 32397, Lusaka, Zambia; 3 Department of Food Safety and Infection Biology, Norwegian School of Veterinary Science, PO Box 8146, Dep, 0033 Oslo, Norway; 4 National Veterinary Institute, PO Box 8156 Dep, N-0033 Oslo, Norway

## Abstract

**Background:**

Bovine brucellosis caused by *Brucella abortus *is endemic in most large commercial and smallholder cattle farms of Zimbabwe, while brucellosis in other domestic animals is rare. The diagnosis of brucellosis is mainly accomplished using serological tests. However, some *Brucella *spp. have been isolated from clinical cases in the field and kept in culture collection but their biochemical profiles were not documented. We report biochemical profiling and AMOS-PCR characterization of some of these field isolates of *Brucella *originating from both commercial and smallholder cattle farming sectors of Zimbabwe.

**Findings:**

Fourteen isolates of *Brucella *from culture collection were typed using biochemical profiles, agglutination by monospecific antisera, susceptibility to *Brucella*-specific bacteriophages and by AMOS-PCR that amplifies species- specific IS711. The results of the biochemical profiles for *B. abortus *biovar 1 (11 isolates) and biovar 2 (2 isolates) were consistent with those of reference strains. A single isolate from a goat originating from a smallholder mixed animal farm was identified as *B. melitensis *biovar 1. The AMOS-PCR produced DNA products of sizes 498 bp and 731 bp for *B. abortus *(biovar 1 and 2) and *B. melitensis *biovar 1, respectively.

**Conclusion:**

We concluded that the biochemical profiles and AMOS-PCR characterization were consistent with their respective species and biovars. *B. abortus *biovar 1 is likely to be the predominant cause of brucellosis in both commercial and smallholder cattle farms in Zimbabwe.

## Background

The Brucellae are small Gram-negative coccobacilli bacteria affecting both animals and humans [1,2]. There are nine recognized species; *B. abortus, B. melitensis, B. suis, B. canis, B. ovis, B. neotomae, B. ceti, B. pinnipedialis *and *B. microti *[3-5]. On the basis of phenotypic profiles, some *Brucella *spp. are further divided into biovars [3].

Studies of the genome of *Brucella *spp. have demonstrated the existence of more than 70% homology [6] and based on DNA-DNA hybridisation, a single species, *B. melitensis *was once proposed, with the other species being biovars [7]. However, the traditional classification into nine different species is still used. This has been further supported by the recent discovery of *B. ceti *and *B. pinnipedialis *from marine mammals [5], or *B. microti *from a common vole *Microtus arvalis *[4]. The genomic similarity makes the differentiation of *Brucella *spp. difficult, and often a study of biological and physiological characteristics is required [3].

In Zimbabwe, only *B. abortus *and *B. melitensis *have been reported to cause animal brucellosis. *B. melitensis *infection was confirmed in a goat flock, believed to have been translocated from Mozambique [8,9]. Brucellosis in wildlife has been demonstrated by serology [10] and in one instance *B. abortus *was isolated from a Cape buffalo (*Syncerus caffer*) [11]. Bovine brucellosis is a problem in some commercial dairy cattle farms, while others have eradicated [12]. Previous studies showed higher seroprevelance of between 10-53% in commercial herds in different regions of the country compared to 0-16% in communal (smallholder) cattle [9,12]. The disease continued to be closely monitored by the use of the milk ring test (MRT), serological surveys and bacteriological investigations [12-14]. Consequently, from 1988 to 2006, *Brucella *spp. isolates have been collected from infected herds from different parts of the country and kept in our laboratories. Although some these isolates have been identified to the species level, the details of their biochemical profiles and biovars have not been documented. The aim of this study was to characterize all *Brucella *field isolates in our culture collection that originated from both commercial and smallholder cattle farms of Zimbabwe using biochemical profiles and polymerase chain reaction (AMOS-PCR). The PCR assay is based on the repetitive genetic element, the insertion sequence 711 (IS711), that is unique to *Brucella *spp. For most *Brucella *spp., multiple copies of the IS711 occur at a unique species or biovar-specific chromosomal locus [15].

## Materials and methods

The details of the identity and origin of all the *Brucella *spp. isolates, including the reference strains, used in this study are listed in Table 1. Prior to use, all strains from culture collection were stored either as lyophilized or in a -80°C deep freezer. Lyophilized isolates were re-constituted and cultured in tryptone soya broth (TSB) (Oxoid) and subsequently sub-cultured onto Farrell's medium (Oxoid) and assessed for purity on bovine blood agar (Oxoid). All plates were incubated at 37°C under 10% CO_2_. Isolates were then inoculated into TSB with 5% glycerol, frozen and exported to Norway for further characterization.

**Table 1:** *Brucella *isolates used in the study and the details of their geographical regions of origin.

**Field *Brucella *spp**.	Reference number (Year isolated)	Specimen of origin	Farm name (type) and geographical region of origin
*B. abortus*	B1-2-2676 (1994)^a^	Aborted foetus	Mazowe (S), MC
*B. abortus*	B4-11-438 (1998)^a^	Aborted foetus	Mhuri (S), MD
*B. abortus*	B5(1999)^b^	Hygroma	Chinamhora (S), MSE
*B. abortus*	B6-304 (1997)^a^	Aborted foetus	Pilosoff (C) MN
*B. abortus*	B7-307 (1997)^a^	Aborted foetus	Chikurubi Prisons (C) MSE
*B. abortus*	B8-2160 (1996)^a^	Aborted foetus	Greyling (C) MW
*B. abortus*	B9-2260 (1996)^a^	Aborted foetus	Hensman (C) MW
*B. melitensis*	B10-6419(1988)^a^	Aborted foetus(goat)	Muzarabani (S)MC
*B. abortus*	B12-gl-55(?)^a^	Aborted foetus	(C), NE
*B. abortus*	B14-(2005)^b^	Milk	Mulanjeni (S), MD
*B. abortus*	B15-H-56(?)^a^	Aborted foetus	NE (C), NE
*B. abortus*	B16-494-64 (?)^a^	Aborted foetus	NE (C), NE
*B. abortus*	B20- (2006)^b^	Milk	Lulaka (S), MD
*B. abortus*	B21-93-35 (?)^a^	Aborted foetus	NE (C), NE

Reference *Brucella *spp.			
*B. abortus *1	544	-	NVI
*B. abortus *2	86/8/59	-	NVI
*B. abortus *3	Tulya	-	NVI
*B. abortus *4	292	-	NVI
*B. melitensis *1	16M	-	NVI
*B. melitensis *3	Ether	-	NVI
*B. suis *1	1330	-	NVI
*B. suis *4	40	-	NVI
*B. canis*	RM-6/66	-	NVI
*B. ovis*	63/290	-	NVI
*B. neotomae*	5K-33	-	NVI

^a^Obtained from the Central Veterinary Laboratory

^b^Obtained from the University of Zimbabwe

(?) = Year of isolation not established, (S) = smallholder farm, (C) = Commercial farm, NE = not established, MC = Mashonaland Central province, MSE = Mashonaland East province, MD = Midlands province, MW = Mashonaland West province, MN = Matabeleland North province NVI = National Veterinary Institute, Norway

For the observation of colonial morphology, *Brucella *spp. isolates were cultured on Mueller-Hinton agar (Oxoid) and single colonies were examined using a low power stereoscopic microscope illuminated by obliquely reflected light as described [3]. The tests for production of urease, catalase, oxidase, H_2_S and indole; sensitivity to dyes (thionin and basic fuchsin) were carried out as described by Alton and co-workers [3]. Further tests for CO_2 _requirement, sensitivity to dyes, lysis by bacteriophages (Tbilisi, Tb; Berkeley, Bk_2_; Firenze, Fi; Izatnagar, Iz1; R/C) and agglutination by A, M and R monospecific antisera were carried out at the Central Veterinary Laboratory, UK, using the procedures described [3].

The *Brucella *spp. isolates were grown on Farrell's agar (Oxoid) and incubated for 48 hours at 37°C under 10% CO_2_. To yield DNA, a few colonies from a pure culture were harvested and suspended in 200 μl of sterile distilled water in Ependorf tubes. A homogeneous suspension was made by stirring with the inoculation loop. Bacterial cells were inactivated by heating the tubes at 100°C for 10 minutes on a heating block (Grant Instruments, UK). To separate the DNA, killed bacterial cells were centrifuged at 15, 700 × g for 10 minutes. The supernatant containing crude DNA template was pipetted into new sterile Ependorf tubes and the sediment discarded. The concentration of the extracted crude DNA was measured using a ND-1000 V3.0 spectrophotometer (NanoDrop^® ^Technologies Inc., USA). The DNA was stored at -20°C until further tests were carried out.

The AMOS-PCR (*B. abortus*, *B. melitensis*, *B. ovis *and *B. suis-*Polymerase Chain Reaction) was done as described previously [15] but with minor modifications of the assay environment. Briefly, PCR assay reaction mixture consisted of the following: 1 × PCR buffer (Applied Biosystems), 3 mM MgCl_2_, 200 μM (each) of the four deoxynucleotide triphosphates (dNTPs) (Finnzymes Oy, Espoo, Finland), and the 5 sets of primers (0.2 μM each) of *B. abortus, B. melitensis, B. ovis, B. suis *and IS711-specific primer. One and half units (1.5 U) of AmpliTaq Gold^® ^DNA polymerase (Applied Biosystems) per 45 μl reaction mixture was added and then dispensed into MicroAmp vials (Applied Biosystems). A total of 5 μl DNA template of killed bacteria (estimated at 200 ng/ml) was added per 45 μl reaction mixture. The PCR was performed with a PTC-200 Peltier Thermocycler (Roche Molecular Systems Inc, Almelda, USA). Amplification was performed for 35 cycles, each cycle comprised of denaturation at 95°C for 1 minute and 15 seconds, annealing at 60°C for 2 minutes, and extension at 72°C for 2 minutes. The PCR products were incubated for a further 5 minutes at 72°C to allow elongation of products before storage at 4°C. The PCR products were separated by electrophoresis using 1.5% agarose gel (w/v) (BDH Electran^®^) at 100 V for 1.5 hours. Gels were stained with ethidium bromide and photographed using a gene snap camera (Syngene Pvt Ltd, UK).

## Results

All 14 *Brucella *spp. isolates characterized in this study (Table 1) yielded the following results that are typical of the genus; Gram-negative coccobacilli, non-motile, positive for modified Ziehl-Neelsen staining, oxidase and catalase production, and negative for indole production (Table 2). Their growth on Mueller-Hinton agar produced colonies that were convex, with entire edges and a smooth shiny consistency. The morphological, growth characteristics and biochemical profiles of the field isolates were similar to their respective reference *Brucella *species and biovars (data not shown).

**Table 2:** Basic biochemical and metabolic profiles of field *Brucella *spp. from Zimbabwe.

***Brucella *isolate**	**Biochemical properties**						**Growth on TSA in the presence of dyes**		
	
**reference no**.	**Cat**	**Oxi**	^**a**^**Ure**	**Mot**	**Ind**	**MZN**	**T**_**20**_	**T**_**40**_	**BF**_**20**_
B1	+	+	+	-	-	+	-	-	+
B4	+	+	+	-	-	+	-	-	+
B5	+	+	+	-	-	+	-	-	+
B6	+	+	+	-	-	+	-	-	+
B7	+	+	+	-	-	+	-	-	+
B8	+	+	+	-	-	+	-	-	+
B9	+	+	+	-	-	+	-	-	+
B10	+	+	+	-	-	+	+	+	+
B12	+	+	+	-	-	+	-	-	+
B14	+	+	+	-	-	+	-	-	+
B15	+	+	+	-	-	+	-	-	-
B16	+	+	+	-	-	+	-	-	+
B20	+	+	+	-	-	+	-	-	-
B21	+	+	+	-	-	+	-	-	+

Cat, Catalase; Oxi, Oxidase, Ure; Urea hydrolysis; Mot, Motility test (+ = motile, - = non-motile); Ind, Indole production; MZN, Modified Ziehl Neelsen stain; TSA, Tryptone Soya agar; T_20_, 20 μl/ml thionin; T_40_, 40 μl/ml thionin; BF_20_, 20 μl/ml basic fuchsin; + = positive test result; - = negative test result.

^a^Urea hydrolysis = All isolates positive within 2 hours of culture

Isolates belonging to the same biovars showed consistently similar results, except for their CO_2_-dependence for growth (Table 3). Regardless of the biovar type, seven of the 13 *B. abortus *isolates were CO_2_-independent, while the remaining six strains were CO_2_-dependent. The *B. abortus *isolates were lysed by phages Tb, Fi, Bk_2_, Iz1 and resistant to R/C. Only one isolate was lysed by the R/C phage. The single *B. melitensis *isolate was resistant to all phages but showed partial lysis to Bk_2 _(Table 3). *B. abortus *isolates were agglutinated by A-antiserum and *B. melitensis *by the M-antiserum, but all were not agglutinated by the R-antiserum (data not shown). Eleven and two of the 13 *B. abortus *isolates were identified to be biovars 1 and 2, respectively (Table 3).

**Table 3:** Summary of phenotypic characteristics of the field *Brucella *spp. from Zimbabwe^a^

	Growth characteristics				Monospecific Sera		Phages at RTD					AMOS-PCR	Interpretation
**Isolate No**.	**CO_**2 **_Dependent**	**H_**2**_S**	**BF**	**TH**	**A**	**M**	**Tb**	**BK**_**2**_	**Fi**	**Iz1**	**R/C**	**Size of DNA detected**	

B1, B6, B7, B14, B16, B21	-	+	+	-	+	-	CL	CL	CL	CL	NL	498 bp	*B. abortus *1

B15	-	+	-	-	+	-	CL	CL	CL	CL	CL	498 bp	*B. abortus *2

B10	-	-		+	-	+	NL	PL	NL	NL	NL	731 bp	*B. melitensis *1

B4, B5, B8, B9, B12	+	+	+	-	+	-	CL	CL	CL	CL	NL	498 bp	*B. abortus*1

B20	+	+	-	-	+	-	CL	CL	CL	CL	NL	498 bp	*B. abortus *2

^a ^All tests carried out by the reference laboratory (VLA), Weybridge, UK.

Isolate No. = Isolate identification

DNA: Test by the AMOS PCR

BF = Basic fuchsin at 20 μl/ml (1/50,000 w/v)

TH = Thionin at 20 μl/ml (1/50,000 w/v)

Phages: Tb = Tbilisi, BK_2 _= Berkeley type 2, Fi = Firenze, Iz1 = Izatnagar, R/C = phage lytic for non-smooth species of *Brucella*

CL = Confluent Lysis

PL = Partial lysis

NL = No lysis

RTD = Routine test dilution

+ = positive (yes)

- negative (no)

bp = base pairs

The *Brucella *isolates were detected by the AMOS-PCR and produced predicted amplicons of sizes 498 bp and 731 bp for *B. abortus *and *B. melitensis*, respectively. Similar DNA products were produced for the reference *B. abortus *biovar 1 and *B. melitensis *biovar 1, respectively.

## Discussion

This paper provides the first detailed biochemical profiling and AMOS-PCR characterization of some *Brucella *spp. isolates from Zimbabwe. The phage sensitivity patterns of all the *Brucella *spp. isolates were consistent with what has been reported [16]. However, a single isolate of *B. abortus *(B15) was lysed by R/C phage and this susceptibility could be indicative of the presence of phage attachment sites which are present in the non-smooth phases of brucellae [16]. The *B. melitensis *(B10) also showed an atypical reaction because it was not lysed by the Iz1 phage which normally lyses smooth strains of this species. However, the examination of single colonies on microscopy by Henry illumination [3] showed no sign of dissociation and none of the isolates were agglutinated by the R-monospecific antiserum which is an indicator of dissociation. The agglutination reaction by monospecific antisera showed the predominance of A-specific and M-specific epitopes in our *B. abortus *and the *B. melitensis *isolate, respectively. All smooth strains of *Brucella *may possess either the A, M or both A and M antigenic epitopes on the O chains of the lipopolysaccharides [16].

Although phage typing is used primarily for identification at the nomen species level, some *Brucella *strains, especially *B. melitensis *may show deviation from the standard pattern of susceptibility to Bk_2_, Iz1 and Wb phages [16]. The use of phage typing as a means of differentiating *Brucella *spp. has become less discriminatory as a typing tool because of the discovery of new strains with atypical sensitivity patterns [17].

The growth characteristics and the biochemical profiles of the field *Brucella *spp. isolates (Table 2) were similar to those of the reference strains used in this study. In addition, the results were consistent with what is reported for *Brucella *spp. and biovars [2,3,16]. However, the requirement for CO_2 _for growth was at variance with reports from literature [3]. Although most strains of *B. abortus *biovars 1-4 require CO_2 _for primary isolation, this attribute is quickly lost on repeated subcultures and such isolates will adapt to growing in atmospheres without CO_2 _[3,16].

The use of the AMOS-PCR results were consistent with those reported elsewhere [15]. These results confirmed the identity of the *Brucella *spp. that was obtained using biochemical profiles. The IS711 analysis using AMOS-PCR can identify only three *B. abortus *biovars, 1, 2 and 4; all three biovars of *B. melitensis*; biovar 1 of *B. suis *and *B. ovis*, but the individual biovars within a species are not differentiated [15]. Therefore, further DNA fingerprinting methods such as the variable number of tandem repeat analysis (VNTR) [15] could be used to investigate the molecular epidemiology of these *Brucella *isolates.

Although the *B. abortus *used in this study originated from five of the eight geographical provinces of Zimbabwe (Table 1), it is difficult to conclude on the spatial distribution due to the limited number of isolates used. These isolates could possibly be restricted to one or a few geographical regions of Zimbabwe from where they have spread through movement of infected cattle. A study of more isolates is required to determine the spatial distribution of *B. abortus *in Zimbabwe. However, the predominance of *B. abortus *biovar 1 over biovar 2 suggested that it is the major cause of bovine brucellosis in both commercial and smallholder cattle farms. Another study which used fewer *Brucella *isolates from commercial dairy farms reported similar findings [12]. Although *B. abortus *biovar 2 was also detected and originating from both the commercial and smallholder cattle farms, its distribution could be limited to a few isolated areas. Elsewhere in South Africa biovar 1 had been reported to account for about 90% while biovar 2 contributed 10% of all the *B. abortus *isolates [18]. South Africa, to a large extent, shares similar geographic, climatic and livestock husbandry systems with Zimbabwe. While it is difficult to explain the reasons for the distribution of these *B. abortus *biovars in the cattle farming sectors, this could largely be influenced by movement of infected cattle between farms. Some farms often purchase cattle from other farms for the purpose of improving the genetics of their herds [9] or in the case of smallholder farms, to restock their herds which are continuously lost due to infectious diseases and lack of adequate grazing, especially during the drought seasons.

Despite the relatively few isolates studied, our results suggested that *B. abortus *biovar 3 and indeed other biovars may be rare in Zimbabwe, but this requires further study. *B. abortus *biovar 3 has been infrequently reported in South Africa, East and North Africa, while there seems to be no reports of isolation of the other biovars [2,18]. World wide, in countries where bovine brucellosis is endemic, *B. abortus *biovar 1 is predominant and *B. abortus *biovar 2 occurs less frequently while the other biovars are rare [2,18].

## Conclusion

We concluded that the biochemical profiles and AMOS-PCR characterization were consistent with their respective species and biovars. *B. abortus *biovar 1 is likely to be the predominant cause of brucellosis in both commercial and smallholder cattle farms in Zimbabwe. Further studies are required that will apply DNA-fingerprinting to study distribution patterns of *B. abortus *biovars in Zimbabwe.

## Abbreviations

AMOS-PCR: *B. **a**bortus, B. **m**elitensis, B. ovis, B. **s**uis-*Polymerase Chain reaction; bp: base pairs; DNA: deoxyribonucleic acid.

## Competing interests

The authors declare that they have no competing interests.

## Authors' contributions

GM: Principal investigator, conceived the study, and participated in the design of the proposal, collection of *Brucella *isolates and culture, phenotypic and molecular characterization of isolates, analysis of results and drafting the manuscript. EB: Supervision of *Brucella *isolate collection, participated in phenotypic and molecular characterization of isolates, result interpretation and helped in drafting of the manuscript. JB: Participated in the design of the study, molecular characterization of isolates, result interpretation and drafting of the manuscript. ES: Participated in the design, acquisition of funds and general coordination and helped to draft manuscript. BD: Participated in phenotypic and molecular characterization of isolates, result interpretation, supervision of laboratory work and helped in drafting of the manuscript. All authors have read and approved the final manuscript.
